# Emotional problems across development: examining measurement invariance across childhood, adolescence and early adulthood

**DOI:** 10.1007/s00787-024-02461-3

**Published:** 2024-05-16

**Authors:** Lucy Riglin, Charlotte Dennison, Joanna Martin, Foteini Tseliou, Jessica M Armitage, Amy Shakeshaft, Jon Heron, Kate Tilling, Anita Thapar, Stephan Collishaw

**Affiliations:** 1 Wolfson Centre for Young People’s Mental Health and Centre for Neuropsychiatric Genetics and Genomics, Division of Psychological Medicine and Clinical Neurosciences, Cardiff University, Cardiff, UK; 2 Population Health Sciences and MRC Integrative Epidemiology Unit, University of Bristol, Bristol, UK; 3 Wolfson Centre for Young People’s Mental Health, Cardiff University, Hadyn Ellis Building, Maindy Road, Cardiff, CF24 4HQ UK

**Keywords:** SDQ, Emotional problems, Measurement invariance, MCS, ALSPAC

## Abstract

Emotional problems (anxiety, depression) are prevalent in children, adolescents and young adults with varying ages at onset. Studying developmental changes in emotional problems requires repeated assessments using the same or equivalent measures. The parent-rated Strengths and Difficulties Questionnaire is commonly used to assess emotional problems in childhood and adolescence, but there is limited research about whether it captures a similar construct across these developmental periods. Our study addressed this by investigating measurement invariance in the scales’ emotional problems subscale (SDQ-EP) across childhood, adolescence and early adulthood. Data from two UK population cohorts were utilised: the Millennium Cohort Study (ages 3–17 years) and the Avon Longitudinal Study of Parents and Children (4–25 years). In both samples we observed weak (metric) measurement invariance by age, suggesting that the parent-rated SDQ-EP items contribute to the underlying construct of emotional problems similarly across age. This supports the validity of using the subscale to rank participants on their levels of emotional problems in childhood, adolescence and early adulthood. However strong (scalar) measurement invariance was not observed, suggesting that the same score may correspond to different levels of emotional problems across developmental periods. Comparisons of mean parent-rated SDQ-EP scores across age may therefore not be valid.

**Supplementary Information:**

The online version contains supplementary material available at 10.1007/s00787-024-02461-3.

## Introduction

Emotional problems, including anxiety and depression, are prevalent across development, but different emotional problems show typical onset at different stages of development [1, 2]. Some anxiety disorders such as separation anxiety and specific phobias typically onset in childhood, while social anxiety disorder commonly arises in childhood and adolescence, and agoraphobia, panic disorder and generalized anxiety disorder most commonly manifest in later adolescence or early adulthood. Depression typically onsets during adolescence and early adulthood [3]. Longitudinal population samples spanning childhood and adolescence / early adulthood are required to assess the typical development of emotional problems and help to overcome issues of referral bias in clinical samples.

A barrier to using longitudinal studies to robustly examine change and continuity in emotional problems across different developmental periods is that this requires the use of the same or equivalent measures at each assessment [4]. Moreover, while there is a need for measures that are validated across childhood, adolescence and early adulthood, developmental differences in the presentation of emotional problems mean that it cannot be assumed that measures appropriate for use in childhood are also equally appropriate for use later in development.

One measure commonly used to assess emotional problems in childhood and adolescence is the Strengths and Difficulties Questionnaire (SDQ) [5], which includes an emotional problems subscale (SDQ-EP). The SDQ-EP is a brief screening scale for emotional disorders in children and adolescents, which can be completed by parents, the young person and/or teachers. The items include one somatic item, three anxiety items and one depression item. Self, parent and teacher versions of the SDQ have been validated for children and adolescents, with self-reports suitable for children aged 11 years or older [6–8]. Research in UK cohorts examining the full five-factor model of the SDQ (which includes five subscales, one of which is emotional problems) has shown mixed results regarding the SDQ’s psychometric properties and comparability (invariance) across childhood and adolescence [9, 10]. These studies did not find measurement invariance across the full age ranges examined (age 3/4 to 16/17 years), but it is not clear whether this applies to the SDQ-EP subscale specifically. Moreover, an adult version of the SDQ has been developed using almost identical wording for the SDQ-EP scale, and this subscale has been validated at age 25 years [11]. To our knowledge, it is unknown whether the SDQ-EP subscale specifically captures a similar construct in adolescence and early adulthood as it does across childhood: this is important for studies interested in developmental change and continuity in emotional problems.

The aim of this study was to use two large general population samples in the UK with parent-rated SDQ assessments spanning ages 3 to 17 years (cohort 1) and 4 to 25 years (cohort 2) to examine for the first time, measurement invariance across age in the SDQ-EP. We hypothesised, given developmental differences in emotional problems, that the SDQ-EP would not show measurement invariance by age: while all five subscale items would load onto one underlying factor at each age, the extent to which items load onto an underlying factor would vary with age. We also investigated measurement invariance by rater (self- versus parent-rated) where these data were available in adolescence and early adulthood.

## Method

### Samples

#### Millennium cohort study

The Millennium Cohort Study (MCS) is a prospective UK national birth cohort of children born between 1st September 2000 and 11th January 2002 in England, Wales, Scotland and Northern Ireland. The sample was recruited to be representative of the total UK population and contains 18,552 families (18,827 children) at baseline [12]. Subgroups of the population were intentionally over-sampled (including children living in disadvantaged areas, children of ethnic minority backgrounds and children growing up in the devolved nations of the UK), to ensure that typically hard to reach populations were adequately represented [13]. Where families included multiple births, we included only the oldest sibling. Our primary sample included those with at-least one parent-rated SDQ-EP assessment (any item): *N* = 15,820. The number of participants for whom different number of parental assessments were available (range: 1–6) is shown in Supplementary Fig. 1a. Where self-reported SDQ assessments were available (age 17 years), secondary analyses investigated those with either parent- or self-rated SDQ-EP assessment (any item) at age 17 years: *N* = 10,158.

#### The Avon longitudinal study of parents and children

The Avon Longitudinal Study of Parents and Children (ALSPAC) is another well-established UK prospective birth cohort study. Pregnant women resident in Avon, UK with expected dates of delivery 1st April 1991 to 31st December 1992 were invited to take part in the study. When the oldest children were approximately 7 years of age, an attempt was made to bolster the initial sample with eligible cases who had failed to join the study originally, resulting in a total of 14,901 study offspring alive at 1 year of age [14–16]. Where families included multiple births, we included the oldest sibling. Full details of this study are provided in the Supplementary Information. Our sample included those with at least one parent-rated SDQ-EP assessment (any item): *N* = 11,148. The number of participants for whom different number of parental assessments were available (range: 1–6) is shown in Supplementary Fig. 1b. Where self-reported SDQ assessments were available (age 25 years), secondary analyses investigated those with either parent- or self-rated SDQ-EP assessment (any item) at age 25 years: *N* = 6,060.

### Measures

In both samples, emotional problems were measured using the 5-item emotional problems subscale of the SDQ [5]: often complains of headaches, stomach-aches or sickness; many worries, often seems worried; often unhappy, down-hearted or tearful; nervous [or clingy] in new situations, often loses confidence; many fears, easily scared (text in square brackets was included for ages 3–17 years only, excluded for ages 18 + years). The SDQ is a brief screening questionnaire and was completed by a parent/carer when their children was approximately aged 3, 5, 7, 11, 14 and 17 years in MCS, and at approximately 4, 7, 8, 9, 12, 13, 17 and 25 years in ALSPAC. Self-reported SDQ scores were also collected at approximately 17 years in MCS and 25 years in ALSPAC. Individual items are shown in Table 1. Individual items are scored on a 0–2 scale (0 = not true, 1 = somewhat true, 2 = certainly true). Suggested categories for parent-rated SDQ-EP scores (possible range 0–10) – used here for descriptive purposes only – at ages 4–17 years are: close to average (0–3), slightly raised (4), high (5–6) or very high (7–10) (for self-rated scores: 0–4, 5, 6 and 7–10 respectively) (see https://www.sdqinfo.org).

**Table 1:** Emotional problems unstandardised factor loadings (invariant across age)

	MCS	ALSPAC
1. Often complains of headaches, stomach-aches or sickness	1.00	1.00
2. Many worries, often seems worried	1.36	1.55
3. Often unhappy, down-hearted or tearful	1.27	1.42
4. Nervous [or clingy] in new situations, easily loses confidence	1.32	1.47
5. Many fears, easily scared	1.44	1.65

Factor loadings from the metric invariance models. MCS = Millennium Cohort Study (*N* = 15,820), ALSPAC = Avon Longitudinal Study of Parents and Children (*N* = 11,148). Square brackets for ages 2–17 years only (excluded for ages 18 + years)

### Analyses

Measurement invariance by age for parent-rated SDQ-emotional subscale was assessed using structural equation modelling to model a latent SDQ-EP factor indexed by the 5 ordinal SDQ-EP parent-rated items. In line with recommendations [4], we evaluated increasingly stringent types of measurement invariance following the procedure outlined and recommended for ordinal variables whereby loading and item thresholds are assessed sequentially [17], as shown in Supplementary Fig. 2: (i) confirming configural invariance – the same pattern of loadings across age (all five subscale items load onto one emotional problems latent factor), (ii) metric (“weak”) invariance – a similar degree of factor loadings across age (the loadings of subscale items onto the emotional problems latent factor are the same at each age), and (iii) scalar (“strong”) invariance – similar item thresholds across age (similar scores on the subscale items relate to similar levels of the emotional problems latent factor across age, or conversely similar levels of the underlying emotional problems construct are required before a rater endorses an item) [17]. While some researchers recommend varying loadings and thresholds together, testing these separately has the advantage of being able to more accurately pinpoint and interpret sources of non-invariance [17]. Finally, in-line with recommendation, we tested for variations in latent means only if scalar invariance was observed [18]. Models were fit using a robust weighted least squares estimator (WLSMV), which estimates factor scores using the regression method, including individuals with partially incomplete data (within or between waves). Model fit was assessed using a variety of indices including the comparative fit index (CFI), root-mean-square error of approximation (RMSEA) and standardized root mean squared residual (SRMR), for which values of ≥ 0.95, ≤ 0.06 and ≤ 0.08 (respectively) are generally considered good fit [19], although we note previous work suggesting that the CFI may incorrectly suggest poor model fit for the SDQ due to low correlations between variables [20]. In-line with recommendations, metric invariance was considered supported where ΔCFI<-0.01, ΔRMSEA < 0.015 and ΔSRMR < 0.03 and scalar invariance supported where ΔCFI<-0.01, ΔRMSEA < 0.015 and ΔSRMR < 0.01 [21].

Secondary analyses investigated measurement invariance in self- versus parent-rated scores at age 17 in MCS and at age 25 in ALSPAC. Model fit and factor loadings for the self-rated SDQ-EP are shown in Supplementary Table 1. Analyses were conducted in Mplus version 8 using Delta parametrization [22].

Sensitivity analyses were conducted using sampling weights for MCS: these are outlined in the Supplementary Material.

## Results

**Fig. 1:**
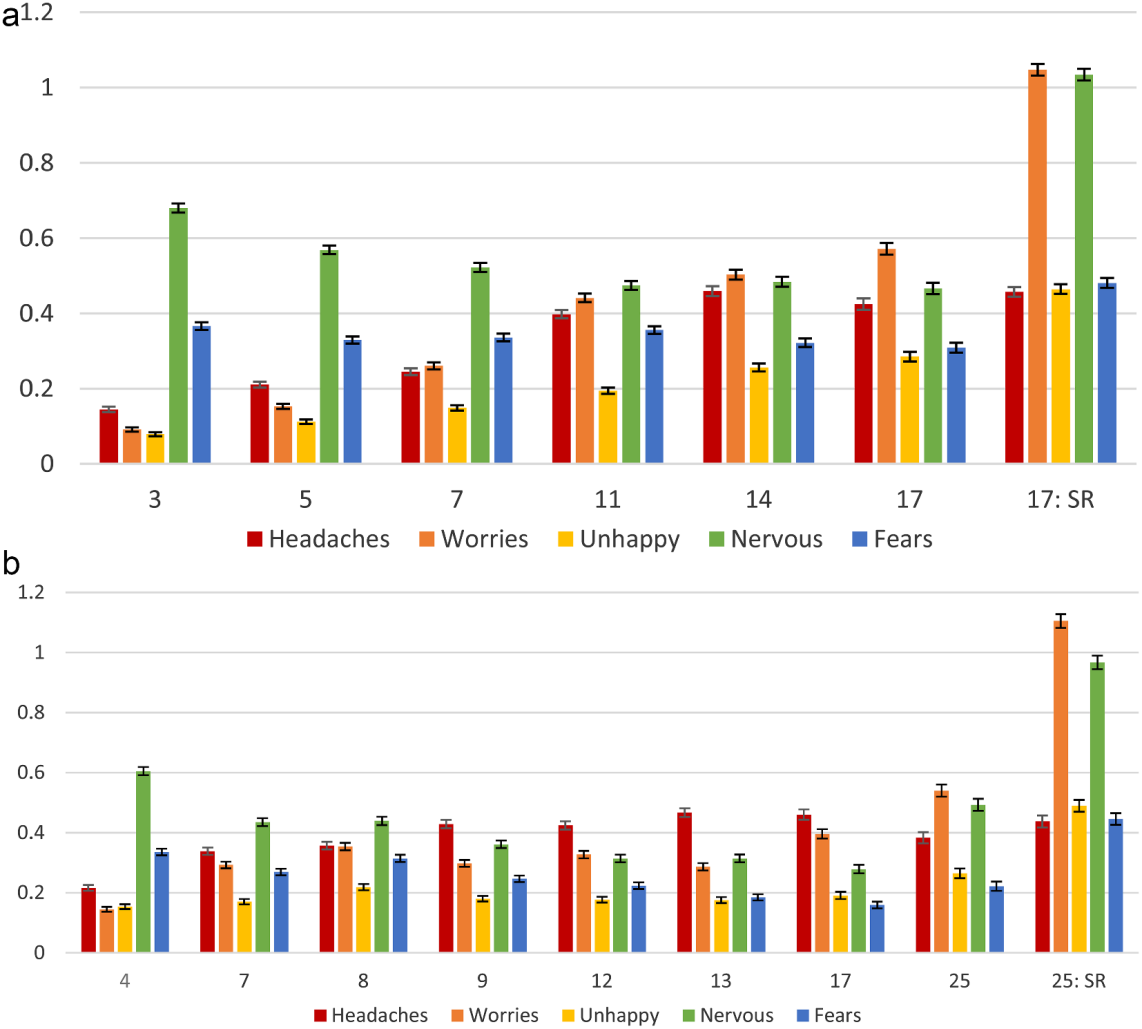
Mean SDQ-EP item score with 95% CI by age, in (**a**) MCS and (**b**) ALSPAC. SR refers to self-report; parent-report unless otherwise stated

SDQ-EP item scores at each available assessment in MCS and ALSPAC are shown in Fig. 1: item scores generally increased over time.

### Millennium cohort study (MCS)

We first fit a single parent-rated SDQ-EP factor in MCS with factor loadings and item thresholds set to freely vary by age (model M1a); this model showed good fit according to the RMSEA and SRMR, but not CFI, providing some evidence of configural invariance (see Table 2). Fixing factor loadings across ages (model M2a) led to an improvement in model fit, providing evidence of metric invariance, but additionally fixing item thresholds by age (model M3a) resulted in decreased model fit (ΔCFI>-0.01 and ΔRMSEA > 0.015), suggesting that the parent-rated SDQ-EP does not show scalar invariance by age (also shown in Table 2). Post-hoc analyses suggested that the SDQ-EP shows scalar invariance by age in early childhood (fixing item thresholds for ages 3 and 5 years only showed limited impact on model fit: ΔCFI<-0.01, ΔRMSEA < 0.015 and ΔSRMR < 0.01), and later in development (fixing item thresholds for ages 11, 14 and 17: ΔCFI<-0.01, ΔRMSEA < 0.015 and ΔSRMR < 0.01) but not across the two (fixing item thresholds for age 7 with younger or older ages ΔCFI>-0.01) (see Table 2). Factor loadings and correlations between factor scores at different ages from the metric invariance model are shown in Tables 1 and 3 respectively; thresholds for this model are shown in Supplementary Fig. 3a: these generally decreased with age for the items relating to headaches, worries and being unhappy, with less clear variation for the items relating to being nervous and having many fears. As scalar invariance was not observed, we did not test for variations in latent means.

**Table 2:** Tests of measurement invariance across age

Model	Free parameters	CFI	RMSEA (90% CI)	SRMR	vs.	Δ parameters	ΔCFI	ΔRMSEA	ΔSRMR	Decision
*Millennium Cohort Study (MCS)*										
M1a: Configural invariance	105	0.894	0.047 (0.046–0.047)	0.068	-			-	-	-
M2a: Metric invariance	85	0.914	0.041 (0.040–0.041)	0.069	M1a	-20	0.020	-0.006	0.001	Accept
M3a: Scalar invariance	35	0.801	0.059 (0.058–0.059)	0.073	M2a	-50	-0.113	0.018	0.004	Reject
Post-hoc analyses: partial scalar invariance from childhood										
Thresholds fixed for age 3–5	75	0.908	0.042 (0.041–0.042)	0.069	M2a	-10	-0.006	0.001	0.000	Accept
Thresholds fixed for age 3–7	65	0.896	0.044 (0.043–0.044)	0.069	M2a	-20	-0.018	0.003	0.000	Reject
Post-hoc analyses: partial scalar invariance from adolescence										
Thresholds fixed for age 14–17	75	0.913	0.041 (0.040–0.041)	0.069	M2a	-10	-0.001	0.000	0.000	Accept
Thresholds fixed for age 11–17	65	0.909	0.041 (0.040–0.042)	0.069	M2a	-20	-0.005	0.000	0.000	Accept
Thresholds fixed for age 7–17	55	0.887	0.045 (0.045–0.046)	0.070	M2a	-30	-0.027	0.004	0.001	Reject
*The Avon Longitudinal Study of Parents and Children (ALSPAC)*										
A1a: Configural invariance	148	0.858	0.047 (0.046–0.047)	0.078	-					
A2a: Metric invariance	120	0.885	0.041 (0.040–0.042)	0.079	A1a	-28	0.027	-0.006	0.001	Accept
A3a: Scalar invariance	50	0.834	0.047 (0.047–0.048)	0.080	A2a	-70	-0.051	0.006	0.001	Reject
Post-hoc analyses: partial scalar invariance from childhood										
Thresholds fixed for age 4–7	110	0.877	0.042 (0.042–0.043)	0.079	A2a	-10	-0.008	0.001	0.000	Accept
Thresholds fixed for age 4–8	100	0.871	0.043 (0.042–0.044)	0.079	A2a	-20	-0.014	0.002	0.000	Reject
Post-hoc analyses: partial scalar invariance from early adulthood										
Thresholds fixed for age 17–25	110	0.880	0.042 (0.041–0.042)	0.079	A2a	-10	-0.005	0.008	0.003	Accept
Thresholds fixed for age 13–25	100	0.875	0.042 (0.042–0.043)	0.079	A2a	-20	-0.010	0.001	0.000	Reject

**Table 3:** Correlations between parent-reported emotional problem latent variables across ages

Millennium Cohort Study (MCS)								
	Age 3	Age 5	Age 7	Age 11	Age 14	Age 17		
Age 3 years	1							
Age 5 years	0.77	1						
Age 7 years	0.62	0.87	1					
Age 11 years	0.46	0.63	0.76	1				
Age 14 years	0.40	0.55	0.63	0.80	1			
Age 17 years	0.34	0.44	0.50	0.67	0.82	1		
*The Avon Longitudinal Study of Parents and Children (ALSPAC)*								
	Age 4	Age 7	Age 8	Age 9	Age 12	Age 13	Age 17	Age 25
Age 4 years	1							
Age 7 years	0.74	1						
Age 8 years	0.66	0.91	1					
Age 9 years	0.55	0.81	0.85	1				
Age 12 years	0.49	0.75	0.75	0.85	1			
Age 13 years	0.42	0.68	0.69	0.77	0.92	1		
Age 17 years	0.35	0.52	0.57	0.58	0.69	0.77	1	
Age 25 years	0.27	0.44	0.46	0.49	0.55	0.60	0.67	1

Estimates from the metric invariance models showing correlations between factors scores

Assessing measurement invariance by rater (self- and parent-report) at age 17 years in MCS using the same procedure suggested acceptable configural and metric invariance, but not scalar invariance (see Table 4), such that thresholds were higher for parent- compared to self-rated items (see Supplementary Fig. 4a). Self- and parent-report factor scores at age 17 years correlated at *r* = 0.58.

**Table 4:** Tests of measurement invariance across raters

Model	Free parameters	CFI	RMSEA (90% CI)	SRMR	vs.	Δ parameters	ΔCFI	ΔRMSEA	ΔSRMR	Decision
*Millennium Cohort Study (MCS) age 17 years*										
M1b: Configural invariance	31	0.944	0.076 (0.073–0.079)	0.058	-			-	-	-
M2b: Metric invariance	27	0.949	0.069 (0.066–0.072)	0.058	M1b	-4	0.005	-0.007	0.000	Accept
M3b: Scalar invariance	17	0.740	0.139 (0.136–0.141)	0.077	M2b	-10	-0.209	0.070	0.019	Reject
*The Avon Longitudinal Study of Parents and Children (ALSPAC) age 25 years*										
A1b: Configural invariance	31	0.973	0.051 (0.047–0.055)	0.051						
A2b: Metric invariance	27	0.975	0.046 (0.043–0.050)	0.052	A1b	-4	0.002	-0.005	0.001	Accept
A3b: Scalar invariance	17	0.766	0.126 (0.123–0.129)	0.074	A2b	-10	-0.209	0.080	0.022	Reject

### The avon longitudinal study of parents and children (ALSPAC)

Examining measurement invariance by age in ALSPAC, for parent-rated SDQ-EP as in MCS, a single SDQ-EP factor with factor loadings and item thresholds free to vary by age (model A1a) showed good model fit according to the RMSEA and SRMR, but again not the CFI, providing some evidence of configural invariance (see Table 2). Fixing factor loadings across ages (model A2a) led to an improvement in model fit, providing evidence of metric invariance, but subsequently additionally fixing item thresholds by age (model A3a) resulted in decreased model fit (ΔCFI>-0.01), suggesting that the SDQ-EP does not show scalar invariance by age. Similar to MCS, post-hoc analyses suggested that the SDQ-EP shows scalar invariance earlier in childhood (fixing item thresholds for ages 4 and 7 years only showed limited impact on model fit: ΔCFI<-0.01, ΔRMSEA < 0.015 and ΔSRMR < 0.01) and later in development (fixing item thresholds for ages 17 and 25 showed limited impact on model fit: ΔCFI<-0.01, ΔRMSEA < 0.015 and ΔSRMR < 0.01) but not across the two (fixing item thresholds for age 7 with older ages or age 17 with younger ages ΔCFI≥-0.01) (see Table 2). Factor loadings and correlations between factor scores at different ages from the metric invariance model are shown in Tables 1 and 3; thresholds for this model showed a similar pattern to MCS (see Supplementary Fig. 3b). Again, as scalar invariance was not observed, we did not test for variations in latent means.

Assessing measurement invariance by rater (self- and parent-report) at age 25 years in ALSPAC using the same procedure suggested acceptable configural and metric invariance, but not scalar invariance (see Table). As in MCS, thresholds were higher for parent- compared to self-rated items (see Supplementary Fig. 4b). Similar to in MCS, self- and parent-report factor scores at age 25 years correlated at *r* = 0.61.

## Discussion

The aim of this study was to examine measurement invariance by age of the parent-rated Strengths and Difficulties Questionnaire emotional problems (SDQ-EP) subscale, using two large UK general population samples with SDQ assessments spanning ages 3 to 17 years, and 4 to 25 years. We found evidence of weak (metric) but not strong (scalar) invariance in both samples.

In both MCS and ALSPAC, we found evidence of weak metric invariance by age, such that at all ages, the five subscale items loaded onto one underlying factor and the extent to which items loaded did not vary by age. This suggests that the basic organisation of the underlying parent-rated SDQ-EP construct is supported across age and that each SDQ-EP item contributes to a latent SDQ-EP construct to a similar degree across these ages. This is not consistent with our hypothesis that there would be variation across development in the extent to which items were indicative of emotional problems. Instead, findings suggest that all items (one somatic, three anxiety and one depression) are similarly indicative of the underlying emotional problems construct captured by this subscale across childhood, adolescence and early adulthood.

However, in both samples findings did not support strong measurement (scalar) invariance, suggesting that the meaning of a given score on the parent-rated SDQ-EP changes across development. Thus, an individual who has the same observed SDQ-EP score in childhood, adolescence and early adulthood may not have the same level of emotional problems across these ages: cut-points for item scores that indicate probable diagnosis may be different at different ages. Conversely, an individual with the same level of underlying emotional problems (as captured by a latent emotional problems factor) in childhood, adolescence and early adulthood is not equally likely to endorse items at these time-points: the “threshold” for endorsing items varies by age. This was particularly the case across transitions from childhood to adolescence and would impact how to interpret developmental changes in parent-rated SDQ-EP scores. The lack of strong (scalar) invariance suggests that comparison of within-person parent-rated SDQ-EP mean scores across age may not be valid: developmental differences in absolute scores therefore need to be interpreted with caution. For example, a decline in absolute parent-rated SDQ-EP scores from childhood to adolescence does not necessarily indicate a decline in emotional problems. In this situation, using age-specific norms is one option for identifying within-individual changes, while accounting for developmental differences (which maybe be due to either measurement non-invariance or genuine developmental differences): this approach has been suggested for the German SDQ-EP [23]. Nevertheless, our finding of weak (metric) invariance suggests that repeated assessments of the total scores for this parent-rated subscale can be used to assess between-person developmental differences from childhood into early adulthood: the subscale can be used to rank participants and then investigate how their rank changes over time. For example comparing groups for whom symptoms decline versus those whose symptoms increase.

We also found weak (metric) invariance by rater at age 17 in MCS and at age 25 years in ALSPAC, suggesting that these self- and parent-ratings of the SDQ-EP items likely capture a similar construct even in late adolescence and early adult life – or at least that the scale items contribute similarly to the underlying subscale construct for each rater. Parent-ratings of emotional problems are not typically used after late adolescence and the switch from parent-reports in childhood to self-reports later in development is often a barrier to robustly examining change and continuity in emotional problems across different developmental periods (Goodman et al., 2007). Indeed agreement between self- and parent-reports is typically modest [24] and this was also true in our samples (factor scores correlated at 0.58 at age 17 years in MCS and 0.61 at age 25 years in ALSPAC). Our findings did not support strong (scalar) invariance, suggesting that mean self- and parent-rated SDQ-EP scores are not equivalent. Specifically, thresholds were higher for parent- compared to self-rated items: higher thresholds indicate that parents endorse items as “somewhat true” or “certainly true” at higher levels of the underlying construct (emotional problems). These findings are consistent with recommended cut-points being lower for parent- rated compared to self-rated SDQ-EP scores (see https://www.sdqinfo.org).

Our findings should be considered in light of several limitations. In particular, both samples, like most longitudinal samples, suffer from non-random attrition, such that individuals with elevated risk of psychopathology are more likely to have dropped out of these birth cohorts by the latest assessments [25–27]. Our inclusion of individuals with missing data will have reduced bias, although this is conditional on data being missing at random with respect to covariates [28]. Our results are strengthened by the use of two independent samples: further replication in additional samples with different patterns of missingness would strengthen these further. Further, we only had early adult data in one sample (ALSPAC) – while an adult version of the SDQ is now available, it has not been used as extensively at this age as it has in childhood and adolescence. As we were unable to assess measurement invariance by age into adulthood in MCS, further work in additional samples at this developmental period is particularly warranted. Finally, in both samples there may have been variation in which parent (or carer) completed the SDQ at each assessment which may have reduced invariance.

In conclusion, we found the parent-rated SDQ emotional subscale to show weak measurement invariance in two large general population samples in the UK, with assessments spanning ages 3 to 17 years and 4 to 25 years. Our findings suggest that items from this subscale load similarly onto one underlying factor across childhood, adolescence and early adulthood. However we did not find strong measurement invariance, suggesting that the same score on the parent-rated SDQ emotional problems subscale may correspond to different levels of emotional problems across developmental periods and that different cut-points may therefore be needed to identify probable disorder at different ages. While this parent-rated subscale may provide useful information about the relative ranking of participants’ emotional problems across development compared to others, mean score comparisons across development will have limited validity. This strengthens the utilisation of this measure in longitudinal research to compare relative developmental change in emotional problems symptoms across groups, at least from childhood to early adulthood. Our findings therefore suggest that the SDQ can be a suitable measure for some but not all research into emotional problems spanning childhood to early adulthood, aiding investigation of these symptoms across developmental periods.

## Electronic supplementary material

Below is the link to the electronic supplementary material.

Supplementary Material 1

## Data Availability

Data access for both samples is through a system of managed open access.

## Code availability

Available on request from the authors.
