# Supplementary material for: Emotional problems across development: examining measurement invariance across childhood, adolescence and early adulthood
Source: Eur Child Adolesc Psychiatry. 2024 May 16;33(12):4237–45. doi: 10.1007/s00787-024-02461-3 (PMC11618155; doi:10.1007/s00787-024-02461-3)
Supplement: Supplementary file 1 — Supplementary Material 1 [file 787_2024_2461_MOESM1_ESM.docx]

**Supplementary Materials**

**The Avon Longitudinal Study of Parents and Children (ALSPAC)**

Pregnant women resident in Avon, UK with expected dates of delivery 1st April 1991 to 31st December 1992 were invited to take part in the study. The initial number of pregnancies enrolled is 14,541 (for these at least one questionnaire has been returned or a “Children in Focus” clinic had been attended by 19/07/99). Of these initial pregnancies, there was a total of 14,676 foetuses, resulting in 14,062 live births and 13,988 children who were alive at 1 year of age. When the oldest children were approximately 7 years of age, an attempt was made to bolster the initial sample with eligible cases who had failed to join the study originally. As a result, the total sample size for data collected after the age of seven is therefore 15,454 pregnancies, resulting in 15,589 foetuses. Of these 14,901 were alive at 1 year of age. Part of this data was collected using REDCap [1, 2]. Ethical approval for the study was obtained from the ALSPAC Law and Ethics Committee and Local Research Ethics Committees. Informed consent for the use of data collected via questionnaires and clinics was obtained from participants following the recommendations of the ALSPAC Ethics and Law Committee at the time. Please note that the study website contains details of all the data that is available through a fully searchable data dictionary and variable search tool: http://www.bristol.ac.uk/alspac/researchers/our-data/. Further details of the study, measures and sample can be found elsewhere [3–5]. Where families included multiple births, we included the oldest sibling.

**The Millennium Cohort Study (MCS): analyses using a sampling weight**

Sensitivity analyses were conducted using a sampling weight in MCS (“weight2”) which weights observations based on representativeness of the UK as a whole. This weight was generated by the MCS for UK-level analysis [6]. Analyses using this weight showed the same patterns of results as the primary analyses (Supplementary Table 2).

**References**

1. Harris PA, Taylor R, Thielke R, et al (2009) Research electronic data capture (REDCap)--a metadata-driven methodology and workflow process for providing translational research informatics support. J Biomed Inf 42:377–381. https://doi.org/10.1016/j.jbi.2008.08.010

2. Harris PA, Taylor R, Minor BL, et al (2019) The REDCap consortium: Building an international community of software platform partners. J Biomed Inform 95:103208. https://doi.org/10.1016/j.jbi.2019.103208

3. Boyd A, Golding J, Macleod J, et al (2013) Cohort Profile: the ’children of the 90s’--the index offspring of the Avon Longitudinal Study of Parents and Children. Int J Epidemiol 42:111–27

4. Fraser A, Macdonald-Wallis C, Tilling K, et al (2013) Cohort Profile: the Avon Longitudinal Study of Parents and Children: ALSPAC mothers cohort. Int J Epidemiol 42:97–110

5. Northstone K, Lewcock M, Groom A, et al (2019) The Avon Longitudinal Study of Parents and Children (ALSPAC): an update on the enrolled sample of index children in 2019 [version 1; peer review: 2 approved]. Wellcome Open Res 4:51. https://doi.org/10.12688/wellcomeopenres.15132.1

6. Plewis I, Calderwood L, Hawkes D, et al (2007) Millennium Cohort Study: technical report on sampling. London Cent Longitud Study, Inst Educ

7. Putnick DL, Bornstein MH (2016) Measurement invariance conventions and reporting: The state of the art and future directions for psychological research. Dev Rev 41:71–90. https://doi.org/10.1016/j.dr.2016.06.004

| **Supplementary Table 1.** Model fit and factor loadings for the self-rated emotional problems subscale | | |
| --- | --- | --- |
|  | MCS  (age 17 years) | ALSPAC (age 25 years) |
| *Model fit* |  |  |
| Free parameters | 15 | 15 |
| CFI | 0.988 | 0.995 |
| RMSEA   (90% CI) | 0.068  (0.061-0.076) | 0.047  (0.036-0.059) |
| SRMR | 0.025 | 0.018 |
| *Factor loadings* |  |  |
| 1. Often complains of headaches, stomach-aches or sickness | 1.00 | 1.000 |
| 2. Many worries, often seems worried | 1.617 | 1.635 |
| 3. Often unhappy, down-hearted or tearful | 1.432 | 1.442 |
| 4. Nervous in new situations, easily loses confidence | 1.319 | 1.318 |
| 5. Many fears, easily scared | 1.445 | 1.522 |
| MCS = Millennium Cohort Study, ALSPAC = Avon Longitudinal Study of Parents and Children | | |

| **Supplementary Table 2.** Tests of measurement invariance in the Millennium Cohort Study (MCS), weighting to the UK population | | | | | | | | | | |
| --- | --- | --- | --- | --- | --- | --- | --- | --- | --- | --- |
| Model | Free parameters | CFI | RMSEA (90% CI) | SRMR | vs. | Δ parameters | ΔCFI | ΔRMSEA | ΔSRMR | Decision |
| *Measurement invariance by age* | | | | | | | | | | |
| M1a: Configural invariance | 105 | 0.893 | 0.040 (0.039-0.041) | 0.070 | - |  |  | - | - | - |
| M2a: Metric invariance | 85 | 0.913 | 0.035 (0.035-0.036) | 0.072 | M1a | -20 | 0.020 | -0.005 | 0.002 | Accept |
| M3a: Scalar invariance | 35 | 0.801 | 0.050 (0.050-0.051) | 0.076 | M2a | -50 | -0.112 | 0.015 | 0.004 | Reject |
| Post-hoc analyses: partial scalar invariance from childhood | | | | | | | | | | |
| Thresholds fixed for age 3-5 | 75 | 0.903 | 0.036 (0.036-0.037) | 0.072 | M2a | -10 | -0.010 | 0.001 | 0.000 | Accept |
| Thresholds fixed for age 3-7 | 65 | 0.894 | 0.038 (0.037-0.039) | 0.072 | M2a | -20 | -0.019 | 0.003 | 0.000 | Reject |
| Post-hoc analyses: partial scalar invariance from adolescence | | | | | | | | | | |
| Thresholds fixed for age 14-17 | 75 | 0.912 | 0.035 (0.034-0.036) | 0.072 | M2a | -10 | -0.001 | 0.000 | 0.000 | Accept |
| Thresholds fixed for age 11-17 | 65 | 0.909 | 0.035 (0.035-0.036) | 0.072 | M2a | -20 | -0.004 | 0.000 | 0.000 | Accept |
| Thresholds fixed for age 7-17 | 55 | 0.888 | 0.039 (0.038-0.039) | 0.073 | M2a | -30 | -0.025 | 0.004 | 0.001 | Reject |
| *Measurement invariance across rates* | | | | | | | | | | |
| M1b: Configural invariance | 31 | 0.940 | 0.070 (0.067-0.073) | 0.060 | - |  |  | - | - | - |
| M2b: Metric invariance | 27 | 0.945 | 0.063 (0.061-0.066) | 0.061 | M1b | -4 | 0.005 | -0.007 | 0.001 | Accept |
| M3b: Scalar invariance | 17 | 0.733 | 0.124 (0.122-0.127) | 0.081 | M2b | -10 | -0.212 | 0.061 | 0.020 | Reject |

|  |
| --- |
| a) Millennium Cohort Study (MCS) |
|  |
| b) Avon Longitudinal Study of Parents and Children (ALSPAC) |
| **Supplementary Figure 1.** Number of time-points individuals had parent-rated emotional problems data available |

| a) Example of configural invariance | b) Example of configural noninvariance |
| --- | --- |
| 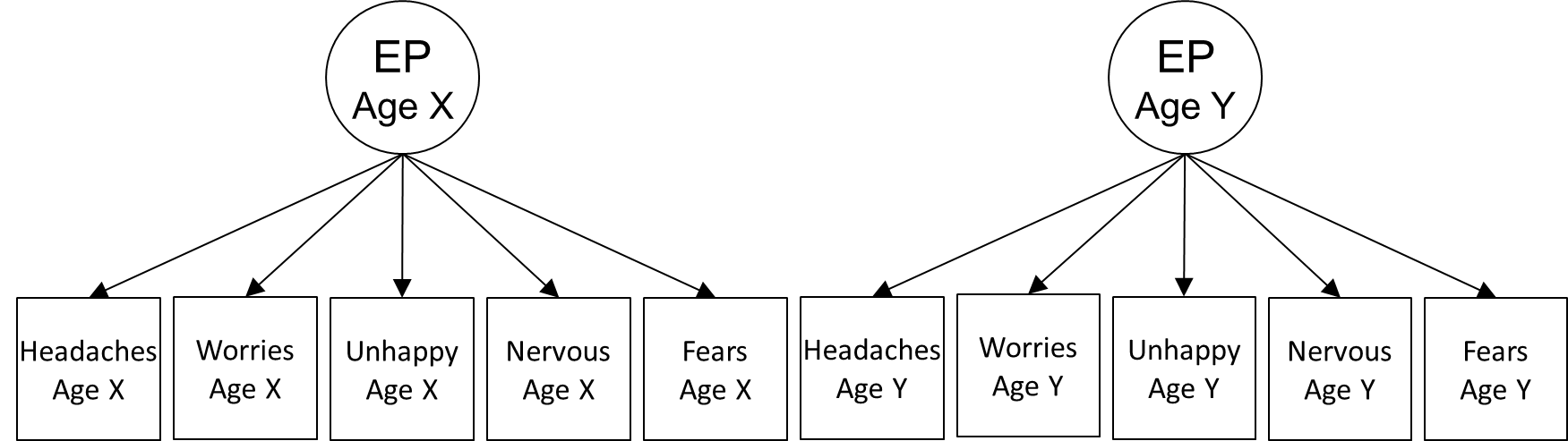 | 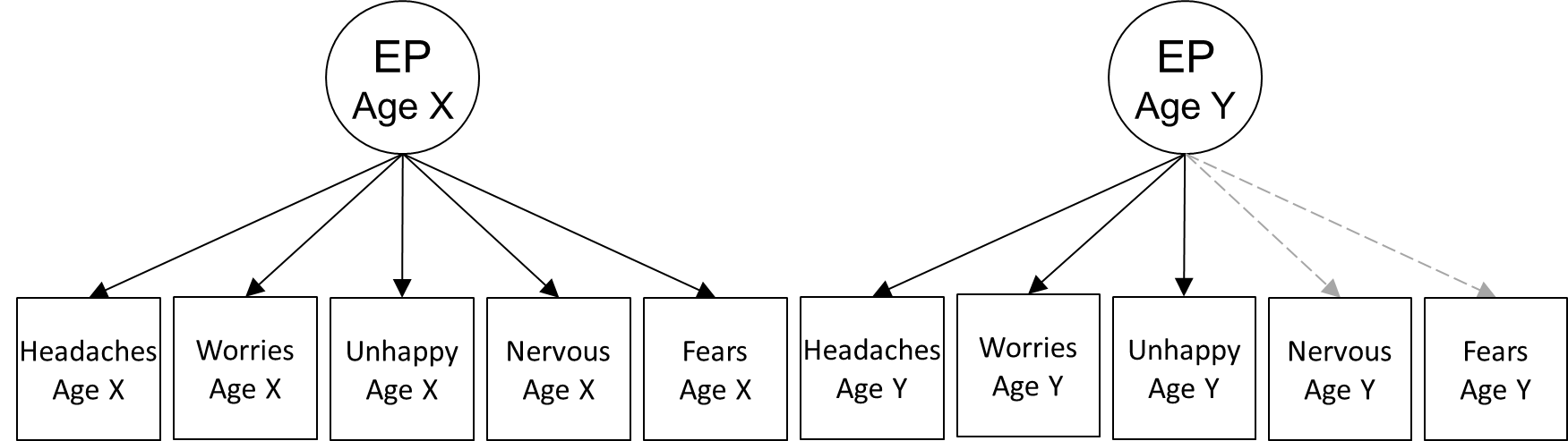 |
| c) Example of metric invariance | d) Example of metric noninvariance |
| 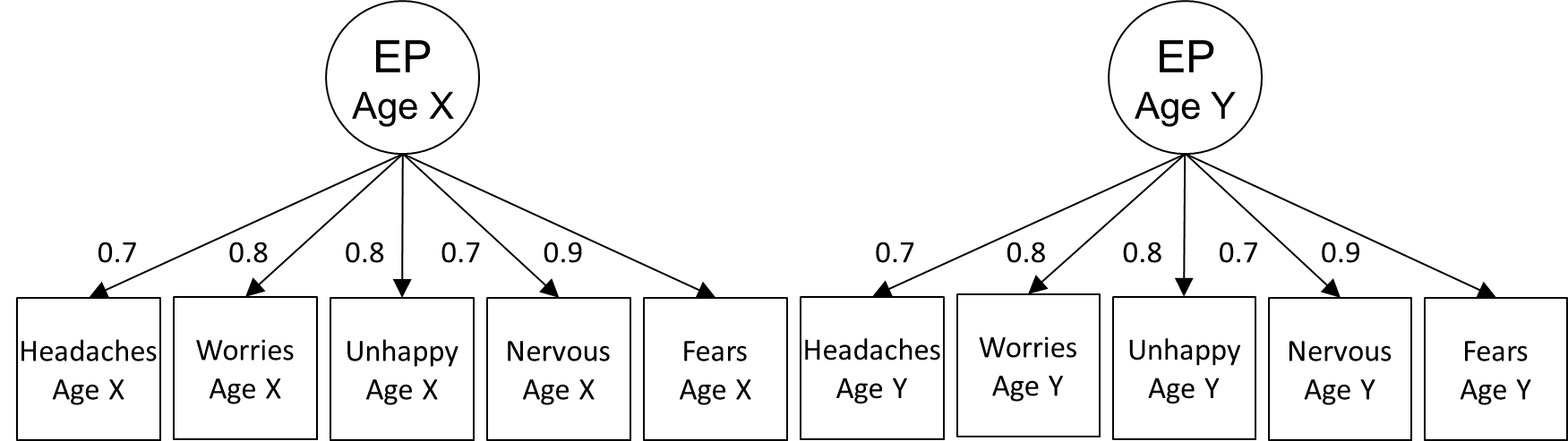 | 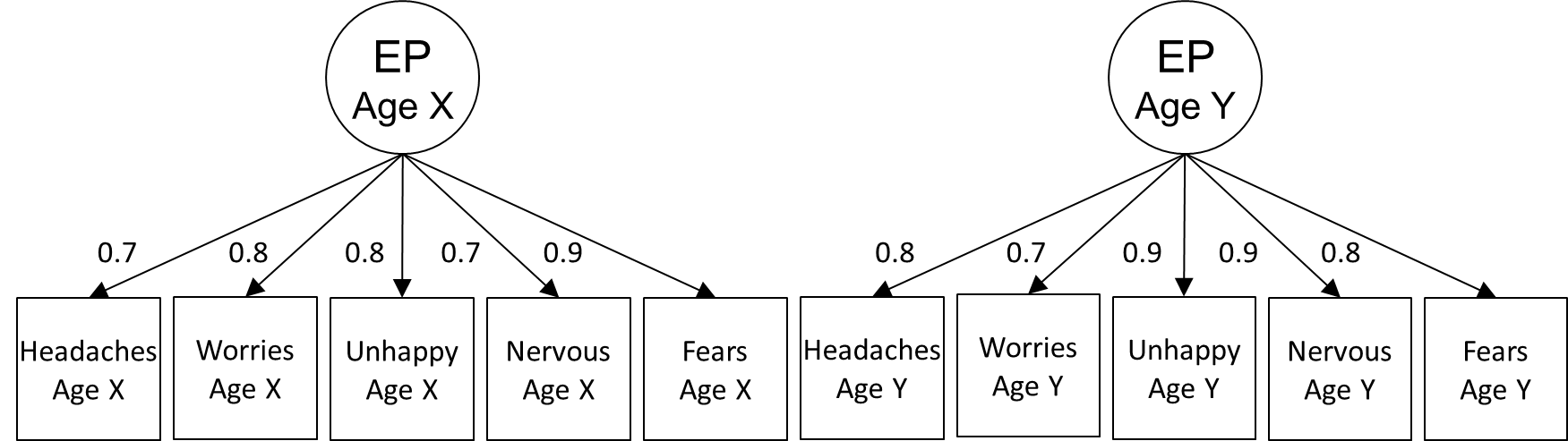 |
| e) Example of scalar invariance | f) Example of scalar noninvariance |
| 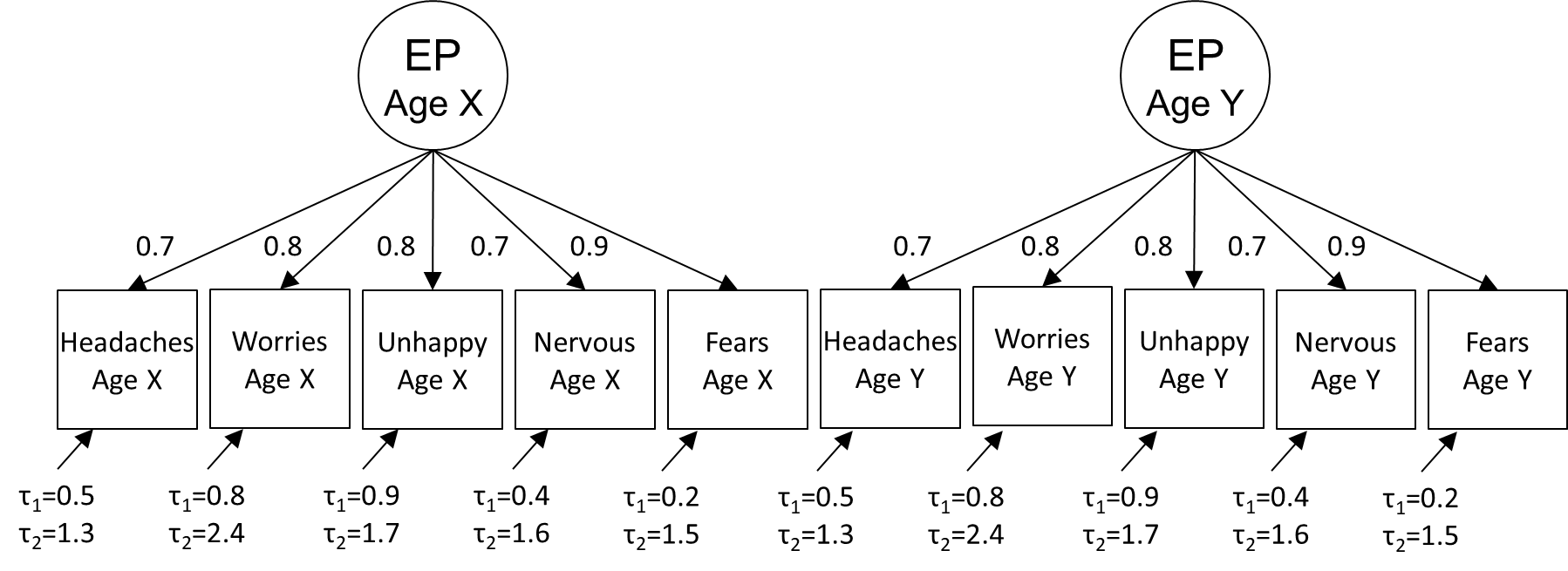 | 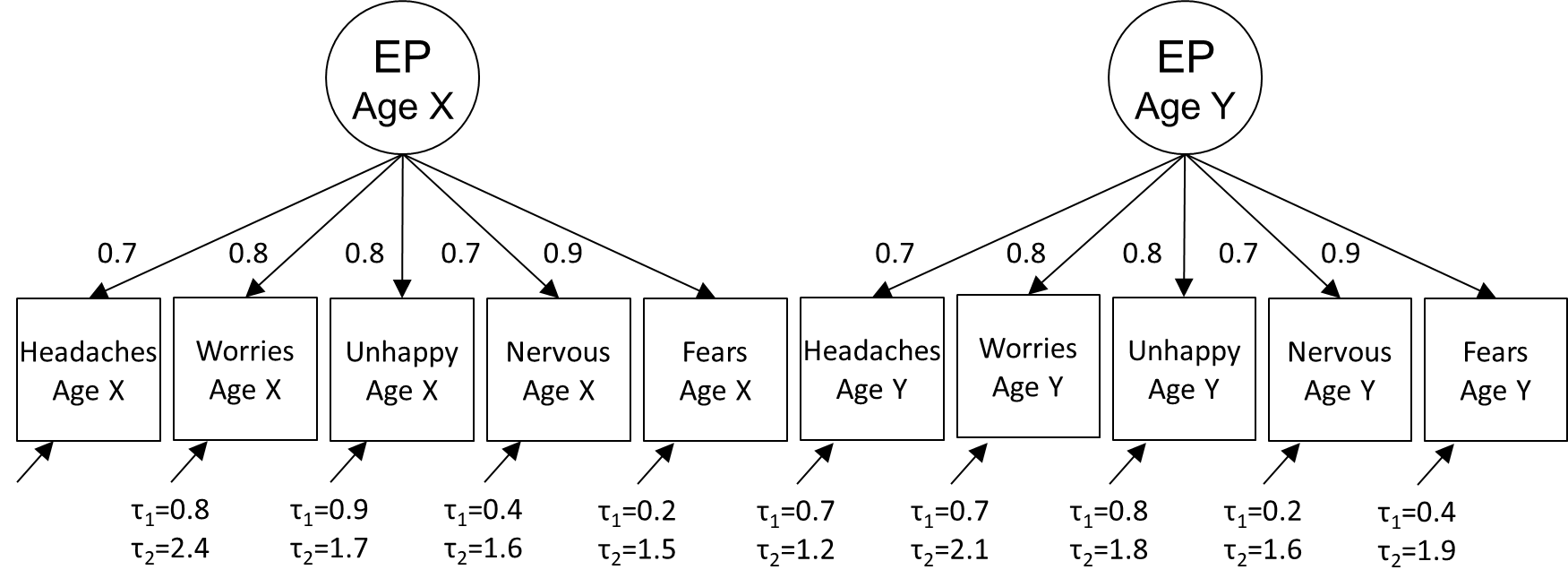 |
| **Supplementary Figure 2**. Examples of configural, scalar and metric (non) invariance, adapted from Putnick & Bornstein (2016). τ=threshold. | |

|  |
| --- |
| **Supplementary Figure 3a**. Thresholds from the parent-rated metric age invariance models for the Millennium Cohort Study (MCS) |
|  |
| *Note.* Thresholds indicate the level of underlying emotional problems (as captured by the latent emotional problems factor) at which a participant endorses an item to be somewhat true (τ_1_) or certainly true (τ_2_). A higher threshold indicates a higher level of underlying emotional problems is required before the item is endorsed. |

|  |
| --- |
| **Supplementary Figure 3b**. Thresholds from the parent-rated metric age invariance models for the Avon Longitudinal Study of Parents and Children (ALSPAC) |
|  |
| *Note.* Thresholds indicate the level of underlying emotional problems (as captured by the latent emotional problems factor) at which a participant endorses an item to be somewhat true (τ_1_) or certainly true (τ_2_). A higher threshold indicates a higher level of underlying emotional problems is required before the item is endorsed. |
|  |
| **Supplementary Figure 4a**. Thresholds from the metric rater invariance models for the Millennium Cohort Study (MCS) |
|  |
|  |
| **Supplementary Figure 4b**. Thresholds from the metric rater invariance models for the Avon Longitudinal Study of Parents and Children (ALSPAC) |
|  |
| *Note.* Thresholds indicate the level of underlying emotional problems (as captured by the latent emotional problems factor) at which a participant endorses an item to be somewhat true (τ_1_) or certainly true (τ_2_). A higher threshold indicates a higher level of underlying emotional problems is required before the item is endorsed. |
